# Amide-type local anesthetics may suppress tumor cell proliferation and sensitize Human Hepatocellular Carcinoma Cells to Cisplatin *via* upregulation of *RASSF1A* expression and demethylation

**DOI:** 10.7150/jca.46630

**Published:** 2020-10-23

**Authors:** Dongtai Chen, Yan Yan, Jingdun Xie, Jiahao Pan, Yonghua Chen, Qiang Li, Yunfei Yuan, Weian Zeng, Wei Xing

**Affiliations:** 1 Department of Anesthesiology, Sun Yat-Sen University Cancer Center, State Key Laboratory of Oncology in South China, Collaborative Innovation Center for Cancer Medicine, Guangzhou 510060, China.; 2 Department of Anesthesiology, Huizhou Municipal Central Hospital, Huizhou 516001, China.; 3 Department of Anesthesiology, Peking University Shenzhen Hospital, Shenzhen 518000, China.; 4 Department of Hepatobiliary Oncology, Sun Yat-Sen University Cancer Center, State Key Laboratory of Oncology in South China, Collaborative Innovation Center for Cancer Medicine, Guangzhou 510060, China.

**Keywords:** amide-type local anesthetics, DNA demethylation, cisplatin, *RASSF1A*, human hepatoma cells

## Abstract

**Background:** It has been reported that local anesthetics are toxic to various types of cells. Furthermore, several local anesthetics have been confirmed to exert demethylation effects and regulate the proliferation of human cancer cells. Our previous findings suggest that lidocaine may exert potential antitumor activity and enhance the sensitivity of cisplatin to hepatocellular carcinoma *in vitro* and *in vivo*. A recent study proved that lidocaine sensitizes breast cancer cells to cisplatin via upregulation of *RASSF1A*, a promotor of tumor suppressive gene (TSG) demethylation. We sought to determine whether amide-type local anesthetics (lidocaine, ropivacaine and bupivacaine) exert growth-inhibitory effects on human hepatoma cells and to determine whether amide-type local anesthetics sensitize human hepatoma cells to cisplatin-mediated cytotoxicity via upregulation of *RASSF1A* expression.

**Methods:** Human hepatoma cell lines HepG2 and BEL-7402 were incubated with lidocaine, ropivacaine and bupivacaine. The viability of local anesthetic-treated cells with or without cisplatin was investigated. Further, we evaluated *RASSF1A* expression after treatment of HepG2 and BEL-7402 cells with three local anesthetics and determined the influence of *RASSF1A* expression on the toxicity of cisplatin to these cells.

**Results:** The viability of HepG2 and BEL-7402 cells was significantly decreased by treatment with amide-type local anesthetics (lidocaine, ropivacaine and bupivacaine). In these cells, the combination treatment with cisplatin and local anesthetics exhibited a stronger reduction in viability. Lidocaine, ropivacaine and bupivacaine promoted a significant increase in RASSF1A expression and a decrease in *RASSF1A* methylation. The combined treatment with both local anesthetics and cisplatin resulted in a significantly lower level of HepG2 and BEL-7402 cell viability than that with singular local anesthetics or cisplatin treatment. Moreover, local anesthetics enhanced the cytotoxicity of cisplatin against HepG2 and BEL-7402 cells, accompanied by an increase in RASSF1A expression.

**Conclusions:** These data indicated that amide-type local anesthetics (lidocaine, ropivacaine and bupivacaine) have growth-inhibitory and demethylation effects in human hepatoma cells. We also found that these amide local anesthetics may enhance the cytotoxicity of cisplatin in human hepatocellular carcinoma cells possibly via upregulation of RASSF1A expression and demethylation.

## Introduction

Recently, an increasing number of studies have shown the potential mechanisms of local anesthetic-induced tumor suppression. Several pathways have been described in the literature. On the one hand, local anesthetics, at high doses, are cytotoxic *in vitro* and *in vivo* 1-4, and on the other hand, they may induce sensitization of tumor cells to chemotherapeutics 1, 5 and heat 6.

Another potential mechanism whereby local anesthetics may influence tumor growth is by interaction with the tumor epigenome 3, 7, 8. Studies have indicated that the silencing of tumor suppressor genes (TSGs) through methylation of their promoters is one of the causes of tumor development 9. As one of the natural covalent modifications of chromatin, defined as epigenomic modifications 10, the silencing of TSGs by DNA methylation induces mechanisms responsible for promoting tumor development, such as metastasis, avoidance of apoptosis, and uncontrolled cell growth or maintaining angiogenesis. In malignant tumors, increased methylation often leads to downregulation of tumor suppressor genes, which is conducive to tumor progression 11.

As an example, methylation of promoter regions of TSGs, such as VHL, CDKN2A and BRCA1, renders them inactive in cancer cells 9, 12. Moreover, DNA methylation may facilitate the mutation of TSGs. The tumor suppressive p53 gene is mutated in more than 50% of solid tumors, and 25% of the mutations result from methylated cytosine to thymine changes in the CpG dinucleotides of this gene 13. Quantitative analysis of DNA methylation profiles for more cancer-related genes also indicates a strong association of TSG hypermethylation with lung cancers 14 and colorectal cancers 15. Specific promoter hypermethylation of these genes is dependent on tumor type 16-18. Unlike genetic alterations, epigenetic gene silencing through DNA hypermethylation is potentially reversible, and this probably can provide new opportunities for the clinical treatment of malignant tumors.

Several local anesthetics have been confirmed to exert demethylation effects and to regulate the proliferation of human cancer cells. Procaine promotes DNA demethylation and inhibits the growth of the human breast cancer cell line MCF-7 16 and of human hepatoma cell lines 3. Recently, a study confirmed the demethylation effect of lidocaine in breast cancer cells and found that the demethylation of Ras association domain family 1A (*RASSF1A*) sensitized breast cancer cells to cisplatin-mediated cytotoxicity 8.

Our previous findings suggested that lidocaine may exert potential antitumor activity and enhance the sensitivity of hepatocellular carcinoma to cisplatin *in vitro* and *in vivo* 1. However, whether other commonly used amide local anesthetics, such as ropivacaine and bupivacaine, have antitumor effects in hepatocellular carcinoma has not been proven. The tumor suppressive gene *RASSF1A* 19-22 has also been shown to be silenced by promoter methylation in hepatocellular carcinoma. Therefore, whether local anesthetics sensitize hepatocellular carcinoma cells to cisplatin via upregulation of *RASSF1A* expression is unclear.

The aim of this study was therefore to test two hypotheses: (i) the amide-type local anesthetics lidocaine, ropivacaine and bupivacaine may exert potential antitumor activity in hepatocellular carcinoma and (ii) the three amide-type local anesthetics may sensitize hepatocellular carcinoma cells to cisplatin-mediated cytotoxicity via upregulation of *RASSF1A* expression.

## Materials and Methods

### Cell Culture and Treatment with Reagents

HepG2 and BEL-7402 hepatocellular carcinoma cells were obtained from the Chinese Type Culture Collection (China). Cells were routinely cultured in high glucose in Dulbecco's modified Eagle's medium supplemented with 10% (v/v) fetal bovine serum (Invitrogen, USA) and maintained at 37 °C in a humidified atmosphere of 5% CO2 plus 95% air. Lidocaine, ropivacaine, bupivacaine, 5-aza-2'-deoxycytidine (DAC, a demethylation agent as a positive control) and cis-diammineplatinum dichloride (cisplatin) were purchased from Sigma-Aldrich (USA).

### Cell Counting Kit-8 (CCK-8) Assay

Cell viability was determined by the Cell Counting Kit-8 assay kit (Dojindo Molecular Technologies, Japan) according to the manufacturer's instructions. HepG2 and BEL-7402 cells were seeded at 3,000 cells per well in 96-well plates and were incubated at 37 °C for 24 h to approximately 85% confluence, and then cells were treated with amide-type local anesthetics (one of lidocaine, ropivacaine and bupivacaine) or cisplatin (alone or in combination) at the indicated concentrations. Cell Counting Kit-8 was added to each well, and the plate was incubated for 3 h at 37 °C. The absorbance value (OD) was read at 450 nm using a spectrophotometer, and the data were analyzed using Softmax Pro software (Molecular Devices LLC, USA). Each experiment was performed in triplicate.

### Methylation Analysis of TSG *RASSF1A*

The DNA methylation was analyzed using MethyLight method 23, to quantify the methylation level of *RASSF1A*, which has been reported to be hypermethylated in breast cancer 24. The methylation status of individual CpG sites in this gene within the promoter region was determined by the sodium bisulfite-sequencing assay as described previously 25. Each experiment was performed in triplicate.

### RNA Extraction and RT-qPCR

Total cellular mRNA samples were extracted from cultured cells using the RNeasy Mini Kit (Qiagen, Valencia, CA, USA). Quantification of *RASSF1A* expression was conducted using the QuantiTect SYBR Green PCR Kit (Qiagen, Hilden, Germany) and an ABI 7900HT Sequence Detector System (Applied Biosystems). The sequences of the primers for *RASSF1A* were as follows: forward primer: 5'-AGCC TGAG CTCA TTGA GCTG-3', reverse primer: 5'-ACCA GCTG CCGT GTGG-3'. All mRNA expression levels were normalized to β-actin (forward primer: 5'-GATG AGAT TGGC ATGG CTTT-3', reverse primer: 5'-GTCA CCTT CACC GTTC CAGT-3'), and the ΔΔCt method was used for relative quantification 26. Each experiment was performed in triplicate.

### Protein Isolation and Western Blot Analysis

Total cellular protein samples were extracted using a radioimmunoprecipitation assay buffer containing a protease inhibitor cocktail (Roche Boehringer Mannheim Diagnostics, Switzerland) and quantified using a bicinchoninic acid protein assay kit (Thermo Scientific) according to the manual. Equal amounts of proteins (40 μg/lane) were separated by 8%~10% sodium dodecyl sulfate-polyacrylamide gel electrophoresis and transferred onto polyvinylidene fluoride membranes (Millipore, USA). The membranes were incubated with primary antibodies at 4 °C overnight and then incubated with horseradish peroxidase-conjugated secondary antibodies at room temperature for 1 h. The blots were detected using an electrochemiluminescence system (Pierce Biotechnology, USA).

### Statistical analysis

Data are shown as the mean ± SEM. To determine differences between three or more means, one-way ANOVA with Bonferroni post hoc tests was performed. A *P* value of < 0.05 was considered statistically significant.

## Results

### Effect of amide-type local anesthetics on cell proliferation

**Figure 1:**
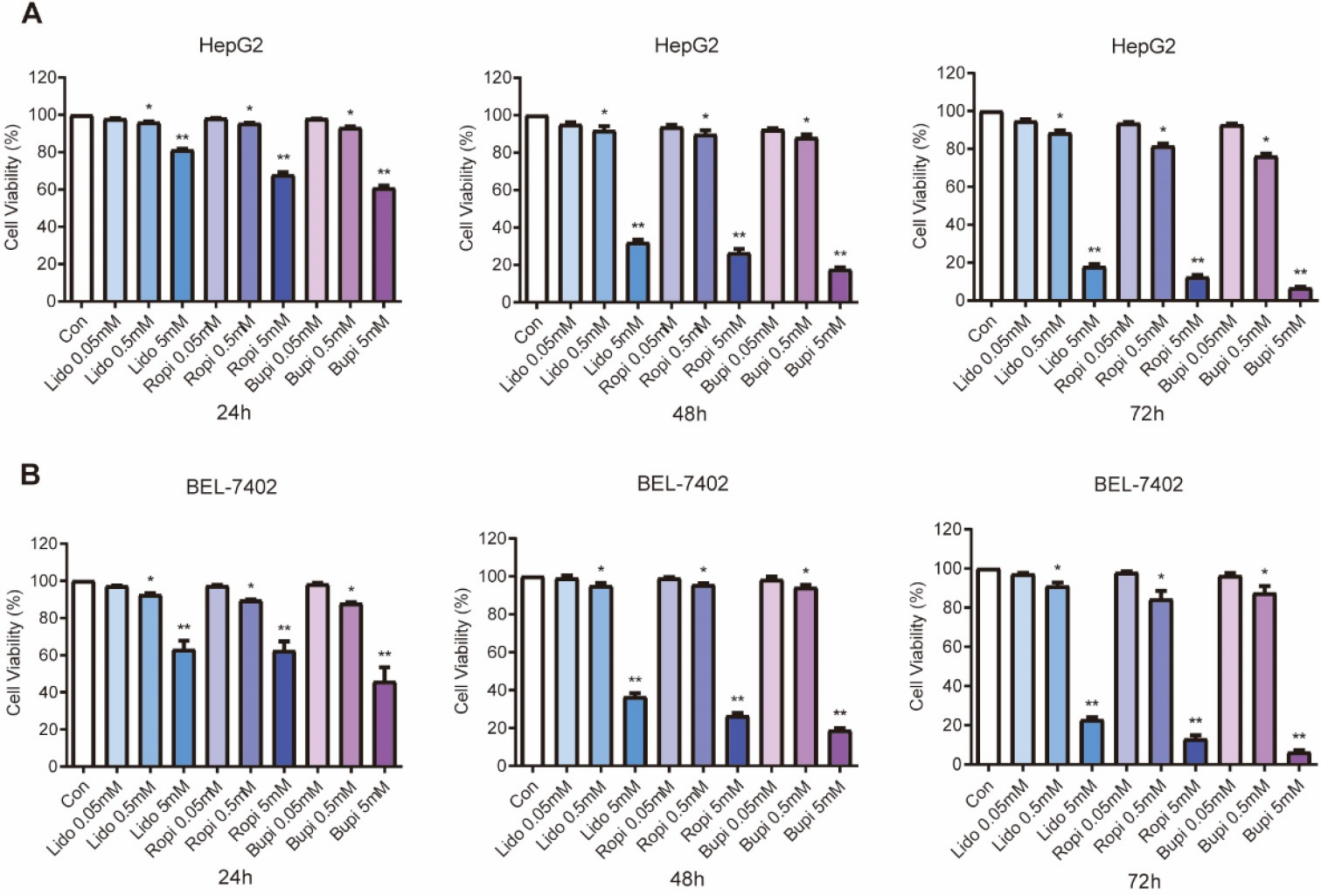
** Effect of lidocaine, ropivacaine and bupivacaine on cell proliferation of HepG2 and BEL-7402 human hepatocellular carcinoma cells.** (**A**) HepG2 cell numbers after lidocaine, ropivacaine and bupivacaine treatment. (**B**) BEL-7402 cell numbers after lidocaine, ropivacaine and bupivacaine treatment. The results are expressed as the mean (SD). Statistical significance is shown as * *P* ˂ 0.05, or ** *P* ˂ 0.01, ns: no significance.

Separate treatment with lidocaine, ropivacaine and bupivacaine at 0.05, 0.5, or 5 mM resulted in significant reductions in cell number, while lower concentrations of local anesthetics had no effect. Anesthetics inhibited the growth of HepG2 (Figure 1A) and BEL-7402 (Figure 1B) cells in a dose-dependent manner.

### Amide-type local anesthetics increase the expression and demethylation of *RASSF1A* gene in HepG2 and BEL-7402 cells

**Figure 2:**
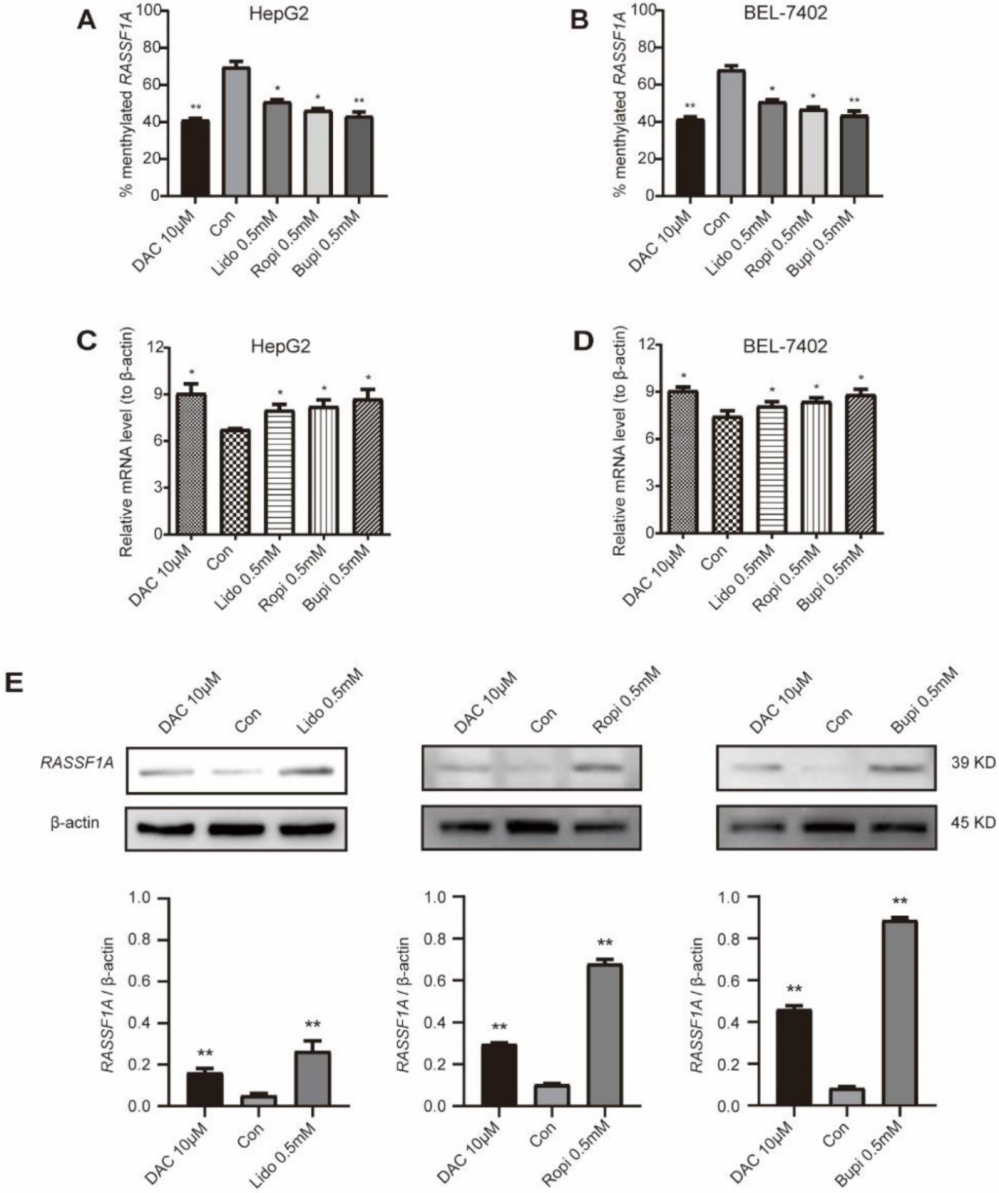
** Local anesthetics upregulate the expression and demethylation of *RASSF1A*. (A)** Methylation levels of *RASSF1A* in HepG2 cells treated for 48 h with 0.5 mM lidocaine, ropivacaine, bupivacaine, or with 10 µM DAC, respectively. **(B)** Methylation levels of *RASSF1A* in BEL-7402 cells treated for 48 h with 0.5 mM lidocaine, ropivacaine, bupivacaine or with 10 µM DAC, respectively. **(C)** mRNA level of *RASSF1A* in HepG2 cells post treatment with 0.5 mM lidocaine, ropivacaine, bupivacaine, or with 10 µM DAC for 48 h, respectively. **(D)** mRNA level of *RASSF1A* in BEL-7402 cells post treatment with 0.5 mM lidocaine, ropivacaine, bupivacaine or with 10 µM DAC for 48 h, respectively. **(E)** Western blot assay showing the protein level of RASSF1A in HepG2 cells with or without 0.5 mM lidocaine, ropivacaine, bupivacaine, or 10 µM DAC treatment for 48 h, respectively. Each value is expressed as the mean (SD) of three independent tests. Statistical significance is shown as * *P* < 0.05, or ** *P* < 0.01, ns: no significance.

Ras association domain family 1A (*RASSF1A*) 28,29, a classical TSG, was found to interact with molecules such as CNK1 33, Nore1 34, MDM2 35, and MAP1S 36 to induce cell death. Methylation of this TSG has been reported to silence its expression and promote the tumorigenesis of hepatocellular carcinoma 19-22 and breast cancers 27-29. We evaluated the methylation levels of *RASSF1A* in HepG2 and BEL-7402 cells with lidocaine, ropivacaine and bupivacaine treatment separately. Figure 2A and B indicates the methylation level of this gene compared to that of the global genomic CpG islands in HepG2 (Figure 2A) and BEL-7402 (Figure 2B) cells; as shown in Figure 2A and B, 10 μM 5-aza-2'-deoxycytidine (DAC, a demethylation agent as a positive control) treatment for 48 h significantly reduced the methylation of the *RASSF1A* gene in HepG2 and BEL-7402 cells compared to that in the control (*P* < 0.05). To examine the influence of promoter demethylation in HepG2 and BEL-7402 cells, the expression of *RASSF1A* mRNA was examined using real-time quantitative PCR (RT-qPCR), and the expression of RASSF1A protein was assessed by western blotting. As shown in Figure 2C and D, there was a significant increase in *RASSF1A* mRNA levels either by 10 μM 5-aza-2'-deoxycytidine (*P* < 0.05) or by 0.5 mM lidocaine, ropivacaine or bupivacaine treatment for 48 h (*P <* 0.05, respectively). Furthermore, the western blot assay reconfirmed the upregulation of RASSF1A at the protein level by demethylation, which was promoted by 0.5 mM lidocaine, ropivacaine or bupivacaine separate treatment for 48 h in HepG2 (Figure 2E; *P* < 0.05) cells. Therefore, the demethylation of *RASSF1A* promotes the expression of this gene.

### Amide-type local anesthetics enhance the cytotoxicity of cisplatin against HepG2 and BEL-7402 cells possibly via upregulating *RASSF1A* expression

**Figure 3:**
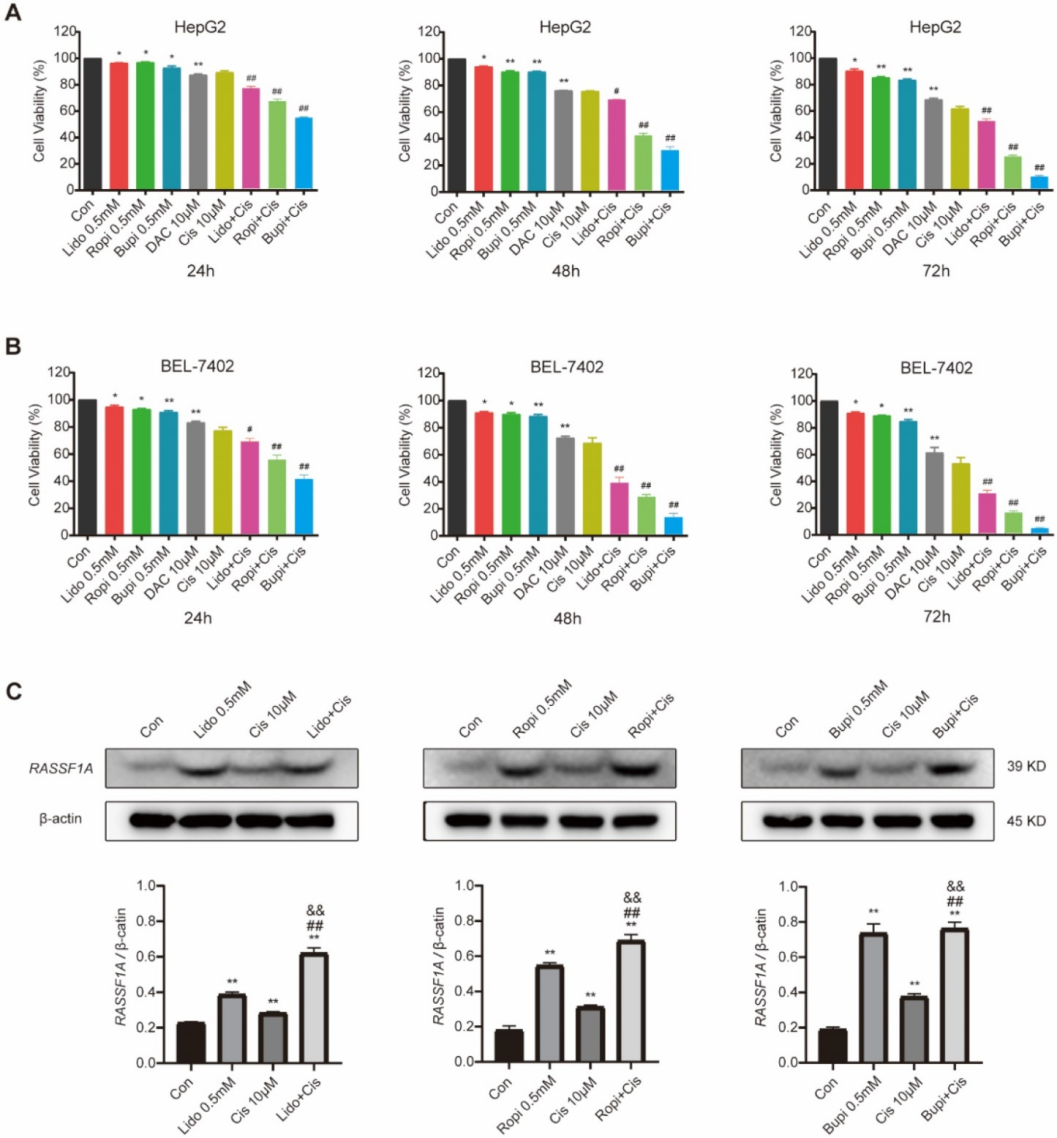
** Local anesthetics enhance the cytotoxicity of cisplatin against HepG2 and BEL-7402 cells possibly via upregulating RASSF1A expression.** (**A**) HepG2 cell numbers after treatment with 10 µM cisplatin or (and) 0.5 mM local anesthetics for 48 h. (**B**) BEL-7402 cell numbers after treatment with 10 µM cisplatin or (and) 0.5 mM local anesthetics for 48 h. (**C**) Western blot analysis of RASSF1A expression in HepG2 cells without or with 0.5 mM local anesthetics and/or 10 µM cisplatin treatment for 48 h. The results are expressed as the mean (SD). Statistical significance is shown as *P < 0.05, or **P < 0.01 different from the corresponding control group; ^&^*P <* 0.05, or ^&&^*P <* 0.01 local anesthetics groups versus local anesthetics plus cisplatin groups; ^#^*P <* 0.05, or ^##^*P <* 0.01 cisplatin group versus local anesthetics plus cisplatin groups; ns: no significance.

Cisplatin has been clinically utilized for hepatocellular carcinoma treatment for decades 27-29, and its antiproliferation effect has been confirmed to be abrogated 30 or enhanced 1, 31 by other molecules. To investigate the regulation of local anesthetics on the cisplatin-mediated antitumor effect, we evaluated the proliferation of HepG2 and BEL-7402 cells by CCK-8 assay after treatment with 0.5 mM of each local anesthetic separately, 10 μM cisplatin, or combinations of cisplatin and each anesthetic. It is shown in Figure 3A and B that HepG2 (Figure 3A) and BEL-7402 (Figure 3B) cells with or without the abovementioned treatment resulted in significant reductions in cell number (*P* < 0.05) and increases in cisplatin cytotoxicity (*P* < 0.01).

To further investigate the association of the local anesthetic-induced expression of RASSF1A with HepG2 cell proliferation inhibition, we analyzed RASSF1A expression in HepG2 cells without or with amide-type local anesthetics and/or cisplatin treatment by western blot assay (Figure 3C). The results showed that the protein expression level of RASSF1A was upregulated when 0.5 mM lidocaine, ropivacaine or bupivacaine were separately combined with cisplatin for 48 h in HepG2 cells (*P* < 0.01).

## Discussion

Studies have indicated that silencing of tumor suppressor genes (TSGs) by promoter methylation contributes to tumor development 9 that the inactivation of TSGs through the hypermethylation of the 5'-CpG islands in gene promoter regions induces processes such as uncontrolled cell growth, metastasis or apoptosis inhibition and also supports the maintenance of angiogenesis 9, 12. As opposed to genetic alteration, epigenetic gene silencing through DNA hypermethylation is potentially reversible, and this presents new opportunities for the clinical management of malignant tumors. DAC (5-aza-2'-deoxycytidine), a demethylating agent, is currently being tested in clinical trials of cancer treatment. DNA demethylation by DAC effectively abolishes 17 β-estradiol-induced cell growth of human breast cancer cells 32; DAC decreases the incidence of gastric cancers and inhibits their growth 33, 34; miRNA-34b inhibits prostate cancer through demethylation, activating chromatin modifications 35. However, the clinical utility of these drugs has not been fully realized because of their adverse effects, such as myelosuppression and mutagenicity 36. The mechanisms of the antitumor effects of DNA demethylation agents still need to be clarified.

Recently, procaine, known as an ester-type local anesthetic, has been revealed to be a nonnucleoside inhibitor of DNA methylation and has a growth-inhibitory effect in human hepatoma cells 3. Lidocaine, known as an amide-type local anesthetic, has been indicated to promote DNA demethylation 8, 37 and increase the cytotoxicity of cisplatin in breast cancer cells via upregulation of *RASSF1A* demethylation 8. In our previous study, we demonstrated that lidocaine may exert potential antitumor activity and enhance the sensitivity of hepatocellular carcinoma to cisplatin *in vitro* and *in vivo* 1.

In the current study, we first used the CCK-8 assay to investigate whether amide-type local anesthetics (lidocaine, ropivacaine and bupivacaine) reduced the viability of human hepatoma cells. Treatment with 0.05, 0.5, or 5 mM lidocaine, ropivacaine or bupivacaine led to a decrease in viable HepG2 and BEL-7402 cells, and the local anesthetics reduced the cell viability in a dose-dependent manner in these cells.

Ras association domain family 1A (*RASSF1A*), a well-recognized tumor suppressive gene in various types of tumors, is a putative tumor suppressor gene located on the 3p21.3 locus 38-40. The hypermethylation of CpG islands in *RASSF1A* occurs in a large percentage of human hepatocellular carcinomas 19-22.

The results here indicate that hypermethylation in this gene was reduced significantly by treatment with three local anesthetics. The three local anesthetics restored the expression of RASSF1A at both the mRNA and protein levels in HepG2 and BEL-7402 cells.

Interestingly, treatment with lidocaine, ropivacaine or bupivacaine for 48 h attenuated the reduction in cell viability promoted by 10 μM cisplatin in both cell lines. The sensitization effect of the three local anesthetics on the antitumor effect of cisplatin was confirmed by CCK-8 assay; combined treatment with 0.5 mM one of the three local anesthetics and 10 μM cisplatin significantly reduced the viability in HepG2 cell lines compared to the singular 0.5 mM one of the three local anesthetics or singular 10 μM cisplatin treatment. Furthermore, we demonstrated by western blot analysis that local anesthetics can enhance the cytotoxicity of cisplatin against hepatoma cells *in vitro* possibly via upregulate of RASSF1A expression.

Although the concentrations of local anesthetics are far too high to be clinically relevant, these concentrations are very similar to those used in previous studies under *in vitro* conditions 37, 41, 42. One of our previous studies also used similar concentrations *in vitro* 1. It is not appropriate to extrapolate our findings here to humans. Nevertheless, our findings suggest the anti-hepatic tumor potential of amide-type local anesthetics.

Taken together, these data indicate that the amide-type local anesthetics (lidocaine, ropivacaine and bupivacaine) have growth-inhibitory effects on hepatoma cells* in vitro*. Each of the three local anesthetics can increase the expression and demethylation of *RASSF1A* gene in HepG2 and BEL-7402 cells. Amide-type local anesthetics may sensitize human hepatocellular carcinoma cells to cisplatin via upregulation of *RASSF1A* expression and demethylation.
